# Simple generation of site-directed point mutations in the *Escherichia coli *chromosome using Red^®^/ET^® ^Recombination

**DOI:** 10.1186/1475-2859-7-14

**Published:** 2008-04-24

**Authors:** Ralf Heermann, Tim Zeppenfeld, Kirsten Jung

**Affiliations:** 1 Ludwig-Maximilians-Universität München, Department Biologie I, Bereich Mikrobiologie, Maria-Ward-Str. 1a, D-80638 München, Germany; 2 Gene Bridges GmbH, Im Neuenheimer Feld 584, 69120 Heidelberg, Germany

## Abstract

**Background:**

Introducing point mutations into bacterial chromosomes is important for further progress in studies relying on functional genomics, systems- and synthetic biology, and for metabolic engineering. For many investigations, chromosomal systems are required rather than artificial plasmid based systems.

**Results:**

Here we describe the introduction of a single point mutation into the *Escherichia coli *chromosome by site-directed mutagenesis without leaving any selection marker. We used Red^®^/ET^® ^Recombination in combination with *rpsL *counter-selection to introduce a single point mutation into the *E. coli *MG1655 genome, one of the widely used bacterial model strains in systems biology. The method we present is rapid and highly efficient. Since single-stranded synthetic oligonucleotides can be used for recombination, any chromosomal modification can be designed.

**Conclusion:**

Chromosomal modifications performed by *rpsL *counter-selection may also be used for other bacteria that contain an *rpsL *homologue, since Red^®^/ET^® ^Recombination has been applied to several enteric bacteria before.

## Background

Red^®^/ET^® ^Recombination is a powerful tool for the chromosomal inactivation of genes or complete operons [1-3]. This method is based on the homologous *in vivo *replacement of a gene/operon of any size and chosen position with a resistance cassette (e.g. a gene conferring antibiotic resistance) in a precise and specific manner by the λ-phage Redγβα recombination system and is applicable to all enteric bacteria. For certain purposes, the introduction of single point mutations is required rather than complete gene replacement or deletion, e.g. to modify a promoter or specifically inactivate the catalytic center of a certain gene product. In most cases, positive phenotypic selection for the introduced mutation is un-achievable, so that mutagenesis must be coupled to a counter-selection approach. A powerful counter-selection system based on the *rpsL *gene (*rpsL*-neo) and streptomycin selection for the introduction of point mutations into Bacterial Artificial Chromosomes (BACs) has been described [4], and this method was further useful for the recombination of large DNA-fragments into BACs [5]. This counter-selection system is based on the *rpsL *gene encoding the S12 ribosomal protein, which is the target of streptomycin. Chromosomal mutations within *rpsL *are responsible for streptomycin resistance [6]. Many *E. coli *strains commonly used for protein overproduction and/or metabolic engineering, including MC4100 [7], JM110 [8], Rosetta DE3 (Novagen, Darmstadt), HB101 (ATCC 33694), and TOP10 (Invitrogen, Karlsruhe) carry an altered *rpsL *gene conferring streptomycin resistance. The counter-selection system takes advantage of the fact that mutations within *rpsL *leading to streptomycin resistance are recessive in a merodiploid strain.

Here we describe a rapid method for site-directed mutagenesis of the *E. coli *chromosome. We used *rpsL *counter-selection in combination with Red^®^/ET^® ^Recombination to introduce a single point mutation into the *kdpA *gene locus of the MG1655 strain genome. We show that *rpsL *counter-selection is applicable for introducing modifications into the bacterial chromosome. Single-stranded synthetic oligonucleotides can be used for recombination, so any chromosomal modification can be designed.

## Results

Introduction of a single point mutation into *kdpA *was chosen to demonstrate *rpsL *based counter-selection in combination with Red^®^/ET^® ^Recombination on the *E. coli *chromosome. KdpA is the K^+^-translocating subunit of the KdpFABC system, a high affinity K^+ ^uptake system in *E. coli*, which is essential for growth under K^+^-limitation [9]. Previously, clones obtained by random mutagenesis were screened for their inability to grow under K^+^-limiting conditions. The mutation in one of these clones (*E. coli *TK2204) was mapped to *kdpA*, and was designated *kdpA4 *[10]. Detailed analysis of *kdpA4 *revealed a point mutation at position 1033 (G to A). This substitution changes glycine 345 to serine. Glycine 345 is located within the K^+ ^selectivity filter of subunit KdpA [11], and the substitution prevents growth under K^+^-limiting conditions. Since *E. coli *strain TK2240 is not isogenic to *E. coli *MG1655 (Tab. 1), and the latter strain was already used for systems biological studies of the Kdp system [12], we aimed to introduce the original *kdpA4 *point mutation into the *E. coli *MG1655 chromosome. As *kdpA4 *prevents growth under K^+^-limitation, no positive selection for the mutants was possible. Thus, we applied the *rpsL *based counter-selection method to screen for mutants.

**Table 1:** *E. coli *strains used and their genotypes

***E. coli *strain**	**Genotype**	**Reference**
MG1655	wild-type; F^- ^lambda^- ^*ilvG rfb50 rph1*	[31]
MC4100	F^-^* araD139 *Δ(*argF-lac*) *U169 rpsL150 relA1 flb-5301 fruA25 deoC1 ptsF25*	[7]
TK2204	F^- ^*thi rha lacZ(am) nagA kdpA4 trkA405 trkD1*	[10]
MG1655 *rpsL150*	F^- ^lambda^- ^*ilvG rfb50 rph1 rpsL150 *(Str^R^)	this work
MG1655 *rpsL150 kdpA4:rpsL-neo*	F^- ^lambda^- ^*ilvG rfb50 rph1 rpsL150 kdpA::rpsL-neo *(Kan^R^, Str^S^)	this work
MG1655 *rpsL150 kdpA4*	F^- ^lambda^- ^*ilvG rfb50 rph1 rpsL150 *(Str^R^) *kdpA4*	this work

In contrast to other K-12 *E. coli *strains (see Introduction), strain MG1655 contains the wild-type *rpsL *gene and is naturally sensitive to streptomycin. Therefore, an altered *rpsL *gene conferring streptomycin resistance was introduced first. For this purpose *rpsL150 *of *E. coli *MC4100 was amplified and recombined into *E. coli *MG1655 carrying plasmid pRed/ET(amp). The temperature sensitive (ts) origin of replication of plasmid pRed/ET(amp) restricts replication at 37°C. After each recombination step cells were incubated at 37°C to remove the plasmid, and the colonies obtained were tested for plasmid loss.

In general, it is not absolutely necessary to cure strains from plasmid pRed/ET(amp) after each recombination step. Since *araBAD *promoter activity is not completely down-regulated in the absence of arabinose, the occurrence of any rearrangement was prevented. Resistant colonies were not obtained on the control plate (cells equally treated without Red^®^/ET^® ^production). The overall recombination frequency was about 4 × 10^-8^/μg DNA. All clones obtained were streptomycin resistant and ampicillin sensitive. The efficiency of proper recombination was 100% (i.e. no false-positives). We observed no differences between *E. coli *MG1655 and *E. coli *MG1655 *rpsL150*, neither in growth nor in *kdpFABC *transcription/KdpFABC translation fidelity (data not shown).

**Figure 1:**
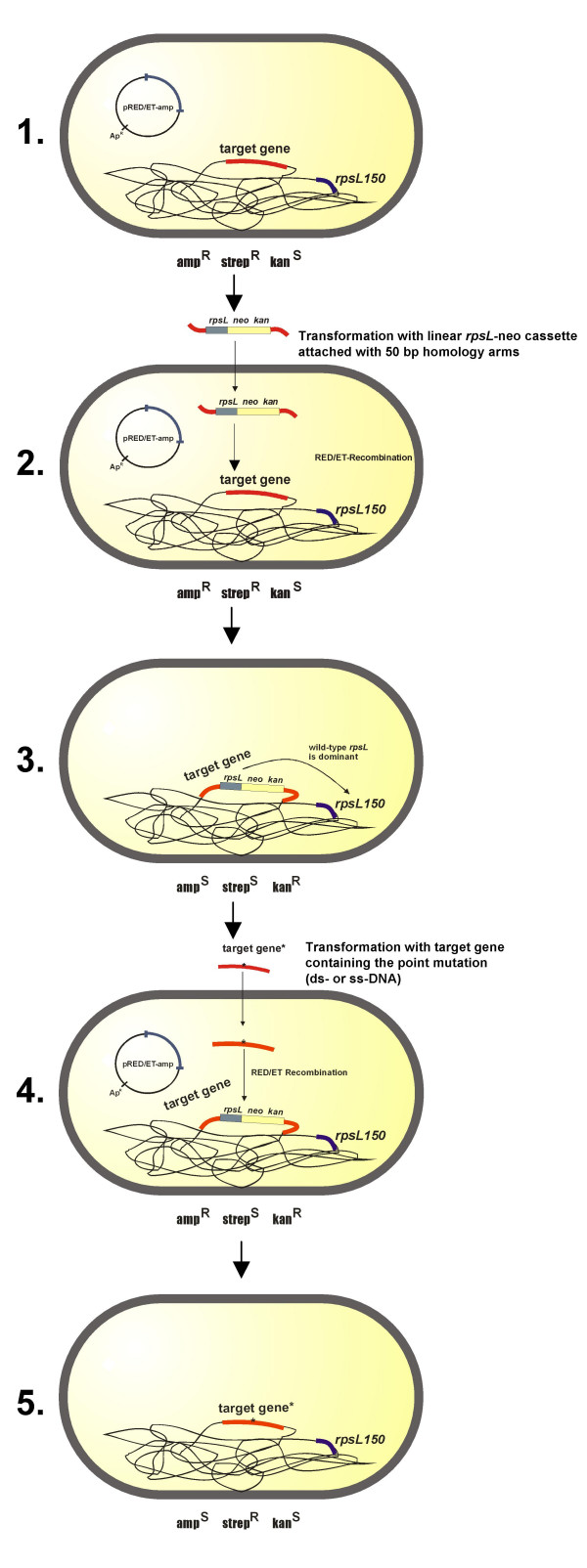
**Basic principle of introducing mutations into the bacterial chromosome using *rpsL*-based counter-selection.** Prerequisite for the used strain is a chromosomal resistance against streptomycin conferred by a mutation in *rpsL*. If necessary, the strain can be made Strep^R ^before by homologous recombination of a mutated *rpsL *gene (e.g. *rpsL150*) (step 1). Around the point of interest within the target gene, the *rpsL*-neo cassette is inserted via 50 bp homology arms by Red^®^/ET^® ^Recombination (step 2). Positive clones are Kan^R^. Due to the additional wild-type allele of *rpsL*, the strain becomes Strep^S ^(step 3). In the next step, the *rpsL*-neo cassette is replaced by Red^®^/ET^® ^Recombination against the modified double-stranded (ds) or single-stranded (ss) DNA-fragment of the target gene (carrying the point mutation) (step 4). Positive clones become Strep^R ^again and can therefore easily be selected (step 5). The asterisk represents the point mutation within the target gene.

The general mechanism of *rpsL *counter-selection is illustrated in Fig. 1. A prerequisite for the strain to be used is a chromosomal encoded resistance to streptomycin conferred by a mutation in *rpsL*, which implies that this method can only be applied to bacterial stains with an *rpsL *homologue. Briefly, cells carrying a mutated chromosomal *rpsL *gene (e.g. *rpsL150*), exhibiting streptomycin resistance, are modified by the introduction of linear DNA comprising the *rpsL*-neo cassette with 50 bp homology arms surrounding the target gene site of interest. The additional wild-type allele of *rpsL *provided by the *rpsL*-neo cassette is dominant over *rpsL150*, and cells become sensitive to streptomycin and resistant to kanamycin, when the cassette has been inserted into the chromosome. In the next step, a DNA-fragment carrying the mutated site in the target gene (e.g. *kdpA*) is introduced into cells, which recombines and replaces the *rpsL*-neo cassette. Due to the loss of the *rpsL *wild-type allele, recombinants regain streptomycin resistance again and can easily be selected. Furthermore, recombinants become kanamycin sensitive due to the loss of the *rpsL*-neo cassette. Selection for streptomycin resistant clones is carried out on complex medium agar plates, so clones grow within 1–2 days of incubation. Within this short incubation time, clones with spontaneous *rpsL *mutations rarely appear.

**Figure 2:**
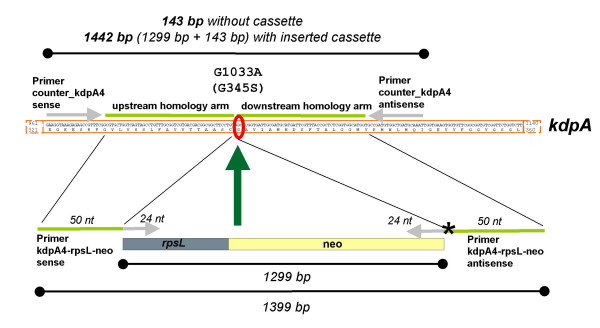
**Primer design to introduce the replacement G1033 to A in *kdpA *encoding KdpA/G345S.** The homologous parts of the homology arms to *kdpA *are indicated as well as the primers for introduction and verification of the *rpsL*-neo cassette inserted into the *kdpA *gene. In the lower part, the composition and the sizes of the linear DNA-fragments for recombination are indicated. The asterisk represents the mutated base in *kdpA*. In the upper part, the sizes of DNA-fragments confirming the introduction or loss of the *rpsL*-neo cassette are indicated. nt = nucleotides.

**Figure 3:**
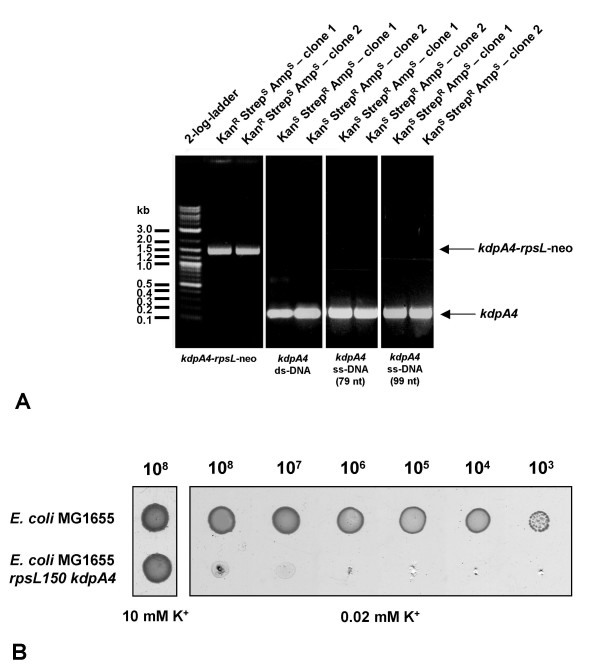
**Verification of the insertion/replacement of the *rpsL*-neo cassette in *kdpA *by PCR (A) and verification of the *kdpA4 *phenotype (growth deficiency under K^+^-limitation) (B).** A Two positive clones of each recombination step were analyzed by colony PCR. On the left panel, insertion of the *rpsL*-neo cassette into *kdpA *was verified, on the two middle and the right panel the replacement of the *kdpA4-rpsL-*neo cassette by the double-stranded and the two single-stranded *kdpA4*-fragments (*kdpA *containing the point mutation G1033A) was confirmed. Below the gels pictures, the respective DNA-fragment used for the recombination is indicated. ds = double-stranded, ss = single-stranded, nt = nucleotides. B Cells were grown overnight in minimal medium containing 10 mM K^+^, washed three times in minimal medium containing 0.02 mM K^+^, diluted, and 2 μl of each suspension containing the indicated number of cells was spotted onto a minimal medium agar plate containing 0.02 mM K^+ ^or 10 mM K^+^, respectively. Cells were incubated overnight at 37°C

Insertion of the *kdpA*G1033A point mutation into the chromosome is illustrated in Fig. 2. The homology arms of the *rpsL-*neo cassette were constructed in a way that a 50 bp homology to *kdpA *was ensured on each side of the point mutation, while the downstream homology arm contained the point mutation. The PCR product *kdpA4-rpsL-*neo (1.4 kb) was recombined into *E. coli *MG1655 *rpsL150 *carrying plasmid pRed/ET(amp). About half of the colonies obtained represented false positives based on the number of colonies observed on the control plates. Plasmid loss appeared to be a strain MG1655 specific problem, because other *E. coli *strains (e.g. HB101, W3110, DH5a, DH10B) generally exhibit >90% validated positives. Nevertheless, the overall recombination frequency for the *kdpA4-rpsL*-neo cassette in *E. coli *MG1655 was about 3 × 10^-7^/μg DNA (25% of clones were correct), which was checked by testing for sensitivity to streptomycin (Strep^S^) and ampicillin (Amp^S^), and resistance to kanamycin (Kan^R^). Introduction of the *kdpA4-rpsL-*neo cassette was verified for two Strep^S^, Kan^R ^and Amp^S ^clones by PCR analysis using counterA4_sense and counter_A4 antisense primers. For both clones, a fragment of 1.44 kb was obtained, which corresponded to the inserted *kdpA4*-*rpsL*-neo cassette (Fig. 2, Fig. 3a).

Next, the *kdpA4-rpsL-*neo cassette was replaced by a DNA-fragment containing the *kdpA4 *mutation, which was obtained by PCR using an *E. coli *TK2204 chromosomal DNA template. The DNA-fragment was recombined into *E. coli *MG1655 *rpsL150 kdpA4:rpsL*-neo carrying plasmid pRed/ET(amp). Depending on the amount of cells plated, limited background growth was visible as a kind of smear at this step. Nevertheless, single colonies were clearly visible. Several hundred clones were obtained, and about half of the number of clones observed on the recombination plate was observed on the control plate. The overall Strep^R^, Kan^S ^and Amp^S ^clone yield was about 10%, indicating that the recombination frequency was about 2 × 10^-7^/μg DNA. The replacement of the *kdpA4-rpsL-*neo cassette against the mutated *kdpA *gene fragment (*kdpA4*) was tested for two clones which were Strep^R^, Kan^S ^and Amp^S ^by PCR analysis. As expected, the size of the PCR products was about 150 bp confirming the loss of the *kdpA4*-*rpsL*-neo cassette (Figs. 2 and 3a).

In principle, this method should work for engineering a mutation into the chromosome using single-stranded synthetic oligonucleotides. To test this, two oligonucleotides were used for recombination, one 79 nucleotides (nt) and one 99 nt in length, consisting of 39 nt and 49 nt length homology arms, respectively, surrounding the point mutation. The recombination frequency for both oligonucleotides was observed to be about 2 × 10^-7^/μg DNA, comparable to that observed for double-stranded DNA. Replacement of the *kdpA4-rpsL-*neo cassette against the mutated *kdpA *gene fragment (*kdpA4*) was confirmed for two Strep^R^, Kan^S ^and Amp^S ^clones from each recombination setup (79 nt and 99 nt oligo) by PCR analysis (Figs. 2 and 3a).

DNA sequencing confirmed the accurate homologous recombination of the fragments within the *kdpA *gene, and introduction of the G1033A mutation. Furthermore, strain MG1655 with the point mutation in *kdpA *was tested phenotypically for growth under K^+^-limitation. As shown in Fig. 3b, cells with a *kdpA4 *mutation exhibit growth defects under K^+^-limiting conditions (0.02 mM K^+^).

## Discussion

New approaches to introduce point mutations into bacterial chromosomes are important for advancing functional genomics and systems biology investigations as well as for metabolic engineering. In this way, mutations are introduced and copy numbers of the encoded proteins are retained. Moreover, it is important to avoid polar effects from remaining antibiotic resistance cassettes in the gene of interest.

Here we demonstrated that *rpsL *counter-selection in combination with Red^®^/ET^® ^Recombination is an efficient approach to modify the *E. coli *chromosome. We introduced a single point mutation into the *kdpA *locus of *E. coli *MG1655, one of the bacterial model strains used widely in systems biological approaches. The method described here is convenient because a selectable marker is used in each step. Furthermore, there is no need for cloning the gene fragment into a special vector, which makes the method time efficient. Recombination between the two *rpsL *alleles is improbable, because Red^®^/ET^® ^Recombination starts recombination at the ends of a linear fragment (Y. Zhang, personal communication), and the wild-type *rpsL *allele located on the linear PCR-fragment is flanked by about 300 bp 50 of which are responsible for precise recombination. The method that we present works with synthetic oligonucleotides as well. Thus it might also be used for the replacement of multiple nucleotides, for deletions or insertions within the gene of interest.

Other counter-selection methods have been described. The introduction of a single point mutation into the *E. coli *chromosome (at the *galK *locus), has been described using λ prophage suicide counter-selection [13]. *thyA- *and *galK-*based counter-selection systems have been successfully applied to the modification of BACs [14,15], and these methods allow modification of DNA without leaving a selectable marker at the modification site likewise *rpsL*-based counter-selection. While the *rpsL *marker gene is only slightly modified, these methods require complete deletion of the respective marker gene. Furthermore, in the case of *rpsL *counter-selection, cells can be grown in complex medium instead of in minimal medium, which speeds up the process and circumvents spontaneous mutations.

Another counter-selection method is *recA- *dependent, which relies on the integration and resolution of a special shuttle vector, and has been successfully applied to modify BACs [16,17]. This method requires time-consuming restriction and ligation steps. These same time-consuming steps are necessary for the "gene gorging" method described previously [18], and most of the other counter-selection systems which use recognition sites for rare-cutting restriction endonucleases, like I-SceI. Nevertheless, these methods were successfully utilized for the modification of BACs, and the *E. coli *genome [19,20]. Recently, counter-selection using I-SceI rare restriction sites has been applied to modify the *Salmonella enteritidis *genome using PCR-based recombination cassettes, thereby overcoming the disadvantages described above [21]. Another common, simple method is *sacB*-based counter-selection [22], but this method implies a high frequency of spontaneous point mutations in the selection marker *sacB*, which significantly increases the background after negative selection [15]. Likewise, spontaneous point mutations within the *rpsL-neo *cassette might also promote the occurrence of false-positive clones. Such false-positive clones can easily be identified by checking for streptomycin sensitivity after recombination of the *rpsL*-neo cassette.

## Conclusion

We present an efficient and non-disruptive approach to introduce point mutations into the *E. coli *chromosome. Chromosomal modifications performed by *rpsL *counter-selection may also be used for enteric bacteria that contain an *rpsL *homologue and in which Red^®^/ET^® ^Recombination is functional. Red^®^/ET^® ^Recombination or analogous methods have been efficiently applied in *Salmonella *[23], *Yersinia *[24], *Shigella *[25], *Citrobacter *[26], and *Serratia *[27] species, and all of these species have a *rpsL *homologue. The versatility of this system illustrates its potential for furthering studies in an important clade of organisms.

## Materials and methods

### Plasmids and Strains

*E. coli *strains are listed in Tab. 1. Plasmid pRed/ET(amp) was obtained from the "Quick and Easy *E. coli *Gene Deletion Kit" (Gene Bridges, Heidelberg), and the *rpsL*-neo template DNA was obtained from the "Counter-Selection BAC Modification Kit" (Gene Bridges, Heidelberg). All oligonucleotides were obtained from Operon GmbH (Köln) in a salt-free grade.

### Media and Growth Conditions

Cells were grown in LB broth [28] under aerobic conditions at the designated temperature. For solid media, 1.5% (w/v) agar was added. Antibiotics were used at the following final concentrations: ampicillin (50 μg/ml), carbenicillin (50 μg/ml in solid media), kanamycin (15 μg/ml), streptomycin (50 μg/ml), and tetracycline (3 μg/ml). For verification of the K^+^-dependent growth deficient phenotype, a phosphate buffered minimal medium was used containing variable amounts of K^+ ^(0.02 mM and 10 mM, respectively) [29].

### Competent cells and transformation

Transformations of cells by introduction of linear DNA-fragments for recombination were performed by electroporation according to the protocol recommended by the technical manual of the "Quick and Easy *E. coli *Gene Deletion Kit" (Gene Bridges, Heidelberg). Briefly, two 1.4 ml cultures of the designated strain were cultivated in 2.0 ml LidBac tubes (Eppendorf, Hamburg) in a thermomixer at 30°C. At an absorbance of 0.3 (600 nm), freshly prepared L-arabinose was added (0.35% w/v, final concentration) to one of the cultures inducing *redγβα/recA *expression, and expression was continued at 37°C. A control culture with no arabinose was incubated as a control. After 45 min, cells were harvested by centrifugation, washed twice with 10% (v/v) ice cold glycerol, and resuspended in a final volume of 30 μl in 10% (v/v) ice cold glycerol. DNA-fragments (400–600 ng ds-DNA, 150 ng ss-DNA) were then added to both samples, and mixtures were incubated on ice for 2 min. Subsequently, samples were transferred to electroporation cuvettes (BioRad, München), and electroporation was carried out with a MicroPulser (BioRad, München) at constant 2.5 kV for ~5 ms (Ec2 program). Cells were immediately removed from the cuvettes by mixing with 1 ml LB medium, and then incubated at 37°C for 3 h. Cells were collected by centrifugation, and all cells (~10^9^) were plated on LB agar containing the appropriate antibiotics.

Transformations of cells by introduction of plasmids were performed with chemically (RbCl) competent cells as described elsewhere [30].

### *In vitro *amplification of DNA-fragments

Linear DNA-fragments comprising either the wild-type or mutated *rpsL *gene (*rpsL150*) were obtained by polymerase chain reaction using primers rpsL_up1 (5'-CTTGACACCTTTTCGGCATCGC-3') and rpsL_down1 (5'-CGTTGTTAATTCAGGATTGTCC-3') with genomic DNA from *E. coli *MG1655 or MC4100, respectively, as templates. The *kdpA4*-*rpsL*-neo cassette was amplified using primers with homology arms consisting of 50 nucleotides upstream and downstream of the targeted point mutation and 24 nucleotides homologous to the *rpsL*-neo cassette (kdpA4-rpsL-neo sense 5'-GCGTGCTGGTCAGTAGCCTGTTTGCGGTCGTGACGACGGCGGCTTCCTGTGGCCTGGTGATGATGGCGGGATCG-3'; kdpA4-rpsL-neo antisense 5'-ACCATGCCACCGAGAGCGGTAAACGAATCATGCATCGCAATCACCGCGCTTCAGAAGAACTCGTCAAGAAGGCG-3') using the *rpsL*-neo template DNA (Gene Bridges, Heidelberg). For amplification of the mutated *kdpA *gene (*kdpA4*), primers that bind upstream and downstream, respectively, of the homology arms were used (counter_kdpA4_sense 5'-AGGTAAAGAGAGCCGTTTCGG-3', and counter_kdpA4_antisense 5'-ATTTGCATCAGCCACATCGGC-3'), and the DNA-fragment was obtained by PCR using genomic DNA of *E. coli *TK2204 as template. For further verification of the point mutation in *kdpA*, the targeted portion of the *kdpA *gene was amplified with primers kdpA2_sense (5'-CCAACGGCGCTGTGCTTTGCC-3') and kdpA2_antisense (5'-GAATAGCGCCAGTTGTTTACG-3'). For all amplifications Phusion™ polymerase (New England Biolabs, Frankfurt) was used, and the protocol recommended for this polymerase was applied (30 cycles, 10 sec 98°C, 20 sec 50°C, 15–30 sec 72°C). For large amounts of DNA, each PCR was performed in parallel several times. PCR products were separated by agarose gel electrophoresis, and DNA was isolated and concentrated by elution in a final volume of 10 μl using the MinElute Gel Extraction Kit (Qiagen, Hilden). For replacement of the *kdpA4-rpsL*-neo cassette by single-stranded oligonucleotides, we used oligonucleotides kdpA4_80_cassette (5'-GTAGCCTGTTTGCGGTCGTGACGACGGCGGCTTCCTGTAGCGCGGTGATTGCGATGCATGATTCGTTTACCGCTCTCGG-3') and kdpA4_100_cassette (5'-GTGCTGGTCAGTAGCCTGTTTGCGGTCGTGACGACGGCGGCTTCCTGTAGCGCGGTGATTGCGATGCATGATTCGTTTACCGCTCTCGGTGGCATGGTG-3') harboring the *kdpA4 *mutation. For verification of correct clones, colony PCR was performed.

## Abbreviations

BAC: Bacterial Artificial Chromosome; nt: nucleotides; ds: double-stranded; ss: single-stranded.

## Authors' contributions

RH has performed the experiments and drafted the manuscript. TZ contributed ideas for performing the experiments, professional support, and helpful suggestions for improving the manuscript. KJ is the project leader and has improved the manuscript. All authors have read and approved the final manuscript.
